# Modality-Resilient Multimodal Industrial Anomaly Detection via Cross-Modal Knowledge Transfer and Dynamic Edge-Preserving Voxelization

**DOI:** 10.3390/s25216529

**Published:** 2025-10-23

**Authors:** Jiahui Xu, Jian Yuan, Mingrui Yang, Weishu Yan

**Affiliations:** School of Optical-Electrical and Computer Engineering, University of Shanghai for Science and Technology, Shanghai 200093, China; 233360797@st.usst.edu.cn (J.X.); 232340583@st.usst.edu.cn (M.Y.); 233360786@st.usst.edu.cn (W.Y.)

**Keywords:** multimodal anomaly detection, cross-modal knowledge distillation, dynamic voxel down-sampling, edge-preserving feature extraction, teacher–student framework

## Abstract

Achieving high-precision anomaly detection with incomplete sensor data is a critical challenge in industrial automation and intelligent manufacturing. This incompleteness often results from sensor failures, environmental interference, occlusions, or acquisition cost constraints. This study explicitly targets both types of incompleteness commonly encountered in industrial multimodal inspection: (i) incomplete sensor data within a given modality, such as partial point cloud loss or image degradation, and (ii) incomplete modalities, where one sensing channel (RGB or 3D) is entirely unavailable. By jointly addressing intra-modal incompleteness and cross-modal absence within a unified cross-distillation framework, our approach enhances anomaly detection robustness under both conditions. First, a teacher–student cross-modal distillation mechanism enables robust feature learning from both RGB and 3D modalities, allowing the student network to accurately detect anomalies even when a modality is missing during inference. Second, a dynamic voxel resolution adjustment with edge-retention strategy alleviates the computational burden of 3D point cloud processing while preserving crucial geometric features. By jointly enhancing robustness to missing modalities and improving computational efficiency, our method offers a resilient and practical solution for anomaly detection in real-world manufacturing scenarios. Extensive experiments demonstrate that the proposed method achieves both high robustness and efficiency across multiple industrial scenarios, establishing new state-of-the-art performance that surpasses existing approaches in both accuracy and speed. This method provides a robust solution for high-precision perception under complex detection conditions, significantly enhancing the feasibility of deploying anomaly detection systems in real industrial environments.

## 1. Introduction

3D anomaly detection, as a pivotal technology in intelligent manufacturing and quality control, has garnered significant attention in recent years. Within industrial visual inspection, traditional approaches predominantly rely on single modalities: either utilising RGB colour images [1] or depending on 3D point cloud data [2]. Methods relying solely on RGB appearance information often struggle with subtle structural defects, while approaches utilising only point cloud geometric information remain insensitive to colour and texture anomalies [3,4,5,6,7,8]. Consequently, integrating colour and shape cues has emerged as a crucial direction for enhancing detection performance, becoming a significant focus of current research.

In this paper, we make a clear distinction between incomplete sensor data and incomplete modality. Incomplete sensor data refers to degraded or partially missing measurements within a given modality. For example, in RGB images, this may appear as motion blur or overexposed regions, while in 3D point clouds, it may manifest as sparse or noisy regions caused by reflective or occluded surfaces. Such cases are directly reflected in public datasets such as MVTec-3D AD, where structured-light scanners often produce incomplete or noisy point clouds due to surface reflectivity or acquisition artefacts. In these cases, the modality itself remains available, but the fidelity of its captured information is compromised. Incomplete modality, on the other hand, denotes the complete absence of an entire sensing channel, such as when RGB images are unavailable but 3D point clouds are still captured (or vice versa). This situation often arises in practice due to sensor failures, cost constraints, or deployment limitations in industrial production lines.

Nevertheless, deploying multimodal anomaly detection on actual production lines presents two major challenges. Firstly, real-world data acquisition environments in industrial settings are often complex and variable, exacerbating the incompleteness and noise inherent in multimodal data. Specular reflections from glossy or metallic surfaces can cause overexposure in certain regions of RGB images, obscuring subtle defects and disrupting local textures. Simultaneously, these reflections interfere with depth sensors, resulting in sparse or noisy point cloud data acquisition [9]. Secondly, the high-speed movement of workpieces on production lines may induce motion blur in RGB images and cause temporal desynchronization in 3D acquisition, a phenomenon exacerbated under low-light conditions [5]. Moreover, sensor lenses frequently become contaminated by dust, oil, or vapour (common in industrial settings such as metal fabrication or packaging) [7], obscuring critical surface features and causing misclassification. Coupled with cost constraints and system complexity, deploying multiple high-resolution sensors at each inspection station is often impractical, compelling systems to adopt selective modal acquisition strategies. These practical challenges make acquiring comprehensive, high-quality multimodal data in industrial settings a significant hurdle. Moreover, processing high-density 3D point cloud data incurs substantial computational overhead. Point clouds of industrial workpieces typically contain millions of points. Unselectively feeding entire models into inference not only causes time-consuming processing that severely impacts production cycles but may also lead to inefficient model training and inference due to redundant points. Consequently, reducing the complexity of point cloud data processing while maintaining detection accuracy remains an urgent challenge [4].

To address the challenges of modality loss and computational overhead, this paper proposes a novel and efficient cross-modal framework for industrial anomaly detection. This framework adopts an integrated design philosophy combining cross-modal pre-training with modality-elastic inference, alongside a 3D feature extraction strategy that incorporates dynamic voxel subsampling and edge retention. First, during training, the teacher model utilises complete RGB and point cloud features to generate high-quality anomaly annotations. The student model learns to retain cross-modal complementary information through a distillation loss (comprising cross-entropy and KL divergence) while randomly missing either modality. During inference, the student model reliably detects anomalies using only available modality inputs, leveraging the cross-modal knowledge acquired during training. This approach of cross-modal pre-training, coupled with modality-deficient, resilient inference, significantly enhances the method’s robustness to incomplete modalities. Secondly, we designed a voxel resolution dynamic adjustment strategy to efficiently extract 3D structural features. Specifically, this strategy adaptively adjusts the voxel size for down-sampling based on local point cloud density, while maximizing the preservation of key geometric edge points to minimize the loss of useful information. This approach substantially reduces the volume of point cloud data and computational cost while maintaining sensitivity to anomalous regions, enabling the real-time processing of large-scale point clouds. Consequently, our method achieves efficient and robust detection of industrial product defects while balancing accuracy and efficiency.

The principal contributions of this work are summarised as follows:

1. Integrated Cross-Modal Pre-training and Missing-Modal Resilient Inference Framework: Proposes a multimodal learning framework for industrial anomaly detection. During training, it fuses RGB and point cloud modalities to learn rich feature representations, while supporting missing-modal inputs during inference. This framework effectively addresses performance degradation caused by incomplete modalities in practical applications, substantially enhancing the robustness of anomaly detection.
2. A dynamic voxel subsampling method with an edge-preserving strategy is designed for highly efficient feature extraction from large-scale point cloud data. It dynamically adjusts voxel subsampling ratios based on point cloud density while extracting structural features that preserve geometric edge information. This approach substantially reduces data volume and computational overhead while maintaining the ability to detect minute defects, laying the foundation for real-time 3D anomaly detection.

Performance Enhancement and Industrial Applicability: The method’s efficacy was validated on public benchmarks, achieving new performance benchmarks on datasets such as MVTec 3D-AD [3], with particularly outstanding results in single-modal inference and high-noise scenarios. Combining high accuracy with high efficiency, it demonstrates strong potential for industrial implementation.

## 2. Related Work

### 2.1. Two-Dimensional Industrial Anomaly Detection

As a key link in quality control, industrial anomaly detection has received extensive attention in recent years. In particular, feature embedding-based methods have shown excellent performance in recent studies.

Feature-embedding-based anomaly detection relies on pre-trained feature extractors (typically visual models trained on large-scale datasets) and specially designed anomaly detection modules [9]. Approaches such as PatchCore [10] and PaDiM [11] identify anomalies by constructing a feature memory bank of normal samples and computing distances between test samples and this memory bank during inference. Methods like SPADE and UniAD further incorporate self-supervised learning strategies, enhancing the discriminative power of feature representations through tasks such as contrastive learning or mask reconstruction [12]. Memory-based approaches have achieved notable success in industrial anomaly detection, offering the advantage of capturing multi-scale semantic and textural features while maintaining low computational complexity.

Leveraging the common principles underlying 2D and 3D anomaly detection, this paper extends memory-based methods to three-dimensional and multimodal settings. By designing a geometry-guided feature fusion mechanism, our approach effectively integrates complementary information from different modalities, significantly enhancing detection accuracy and robustness—particularly when handling small-sized, low-contrast industrial defects. Experimental results demonstrate that the proposed method achieves state-of-the-art performance across multiple industrial anomaly detection benchmarks.

### 2.2. Three-Dimensional Industrial Anomaly Detection

The field of industrial anomaly detection has expanded progressively from two-dimensional to three-dimensional spaces in recent years, addressing the challenges of detecting complex geometries and surface defects. In unsupervised three-dimensional anomaly detection (3D UIAD) for industrial scenarios, mainstream approaches typically utilise raw 3D point clouds or corresponding depth maps as input, aiming to identify and localise anomalous regions through spatial layout, geometric morphology, and depth features. Unlike RGB images, three-dimensional data lacks colour information. Consequently, detection accuracy is often low for minute anomalies, such as surface stains or rust spots, that exhibit no pronounced geometric or abrupt changes. However, for defects with distinct geometric features—such as cracks, holes, or misalignments—3D UIAD methods can achieve more reliable detection outcomes. Currently, the number of UIAD methods that rely solely on pure three-dimensional inputs remains limited.

To address this gap, 3D-ST [1] pioneered the introduction of a student–teacher framework within the three-dimensional point cloud domain. This approach constructs a teacher network specifically tailored for point clouds, pre-training it using local geometric descriptors and supplementary annotation data. This enables the teacher to learn high-dimensional representations of normal geometric structures. Subsequently, the student network learns to extract geometric features from raw point clouds under the guidance of the teacher. Consequently, during inference, accurate detection of geometric anomalies can be achieved using only unannotated point cloud input. Complementing this, Reg3D-AD [13] pioneered the application of a memory bank mechanism in 3D UIAD. It stores feature sets from normal samples within the memory bank, enabling rapid retrieval of similar features through comparative queries. This enables the system to identify whether a given point cloud exhibits geometric anomalies that deviate from the normal distribution, thereby effectively addressing the evaluation challenges posed by insufficient baseline data.

When processing high-resolution point clouds, some studies have further optimised approaches from both feature representation and computational efficiency perspectives. Group3AD [14] proposed a method based on group-level representations, partitioning the input point cloud into several semantic subgroups. It constructs contrastive learning tasks within individual samples, enabling the network to maintain sensitivity to minute geometric changes across different point cloud resolutions and scales. This achieves robust anomaly detection for high-resolution point clouds. In contrast, PointCore [15] focuses on integrating multi-source features within limited computational resources by tightly coupling locally co-occurring features stored in memory with global features extracted by the PointMAE pre-trained model, effectively avoiding the high computational overhead and feature mismatch issues associated with multi-memory schemes.

Additionally, PointCore employs a ranking-based normalisation strategy to mitigate the impact of distribution discrepancies between coordinate anomaly scores and PointMAE anomaly scores for extreme value pairs, thereby balancing the multi-channel anomaly scoring system. Addressing generalization challenges under sparse training data, R3D-AD [16] employs anomaly simulation strategies to generate representative pseudo-anomaly samples by simulating geometric distortions, such as “bulging,” “sinking,” and “damage,” in point clouds. Subsequently, diffusion models reconstruct these pseudo-samples, enabling the detection and localisation of minute anomalies in real point clouds through reconstruction error analysis.

In our research, we adopt a feature embedding-based methodological framework and propose an innovative teacher-student model. Unlike existing approaches that focus solely on local-level cross-modal alignment while neglecting global feature consistency, we design a multi-scale feature fusion mechanism. This simultaneously optimises intra-modal feature compactness and cross-modal information exchange, thereby enhancing anomaly detection accuracy and robustness while maintaining computational efficiency.

### 2.3. Voxel-Based Point Cloud Downsampling

Voxel-based downsampling represents a widely adopted data simplification technique in point cloud processing, originally proposed by Rusinkiewicz and Levoy in 2002 [17]. This method achieves dimensionality reduction and sparsification by partitioning three-dimensional space into a regular cubic grid (voxels) and replacing the original point set with the centroid of each non-empty voxel [11]. The core advantage of voxel subsampling lies in its ability to transform unstructured point cloud data into a regular, discrete representation. This significantly reduces computational complexity in subsequent processing while preserving key features of the original geometric structure [18].

In recent years, researchers have proposed various improved voxel subsampling methods to address the limitations of traditional approaches. Yang et al. introduced an adaptive voxel size-based point sampling method (AVS-Net), which dynamically adjusts voxel size according to the local density distribution of the point cloud, effectively balancing computational efficiency and geometric fidelity [5,9]. Xiao et al. proposed a hierarchical voxel segmentation subsampling method that balances point cloud subsampling effects by increasing the number of segmentation layers, achieving efficient subsampling while preserving key features [19].

Despite voxel down-sampling’s commendable performance with large-scale point cloud data, inherent limitations persist: fixed voxel sizes may cause geometric detail loss, particularly in regions with significant density variations; simple centroid calculations can blur high-curvature features like edges and corners; furthermore, quantisation errors during voxelisation may introduce new noise [20,21,22,23,24,25].

To address the limitations of existing voxel down-sampling methods, an algorithm is proposed that employs a dynamic voxel resolution adjustment strategy for adaptive down-sampling. Its focus lies in preserving critical structural information and detailed textures across different scales. The algorithm comprises two core modules: an adaptive down-sampling unit module and a point cloud contour extraction module. The adaptive subsampling unit module dynamically adjusts voxel parameters based on the local geometric characteristics of the point cloud, whilst the point cloud contour extraction module concentrates on preserving key structural features within the point cloud.

### 2.4. Cross-Modal Knowledge Distillation

Cross-Modal Knowledge Distillation (CMKD), an effective knowledge transfer paradigm, has made significant progress in multimodal learning over the past few years. Traditional multimodal architectures typically require complete modal inputs during both training and inference phases, severely limiting their applicability in practical scenarios involving modal deficiencies or sensor failures. Cross-modal knowledge distillation establishes knowledge bridges between different modalities, enabling models to maintain high performance under single-modal conditions while retaining the advantages of multimodal complementary information.

The theoretical foundation of cross-modal knowledge distillation can be traced back to Vapnik’s Learning Using Privileged Information (LUPI) framework [26], in which the teacher model accesses additional “privileged” modal information, while the student model must reason under conditions where this privileged information is absent. Unlike traditional knowledge distillation, cross-modal knowledge distillation faces the core challenge of domain gaps between modalities. This often renders direct application of unimodal knowledge distillation methods ineffective in cross-modal scenarios.

Recent research indicates that the domain gap manifests primarily in two aspects: modality imbalance and soft label misalignment [27]. The former reflects significant disparities in prediction confidence across modalities for target categories, while the latter reveals divergent prediction distributions for non-target categories. These discrepancies render straightforward imitation of the teacher modality by the student modality both challenging and limited in effectiveness.

In industrial anomaly detection, cross-modal knowledge distillation demonstrates unique advantages. Traditional approaches, such as the hallucination network proposed by Garcia et al., enhance video action recognition by generating RGB-D cross-modal representations. Wang et al.’s LCKD effectively addresses missing modality issues by identifying the most pertinent modality as the teacher [28,29,30,31].

However, existing research has overly focused on completing training for missing modalities to improve performance, while neglecting in-depth exploration of the intrinsic correlations between modalities. Our experimental results demonstrate that studying inter-modal correlations holds greater significance for guiding cross-modal knowledge distillation. Particularly in industrial anomaly detection scenarios, understanding the complementary relationships between different modalities (such as RGB images, depth maps, and thermal imaging) enables more effective utilization of existing modalities in quality control and leveraging human experts’ prior knowledge. This approach ultimately enhances the defect identification rate in multimodal industrial anomaly detection.

## 3. Methodology

### 3.1. Overview

**Figure 1:**
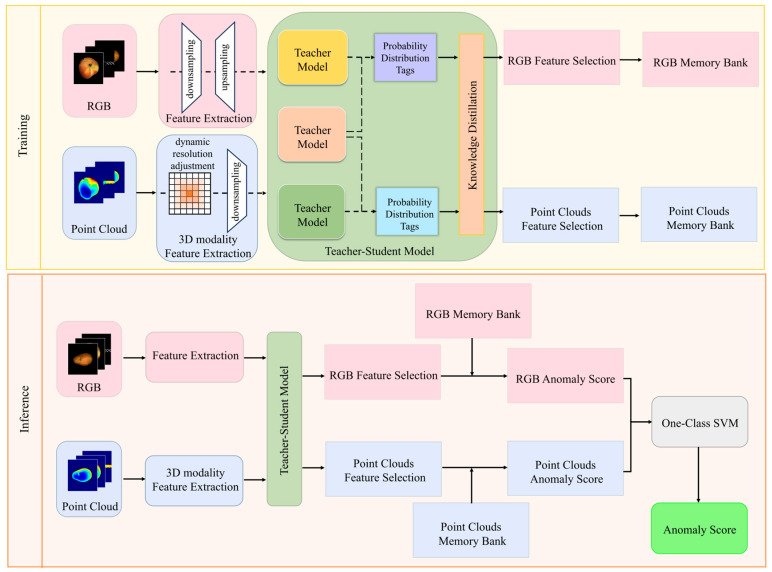
Dual-modal complementary learning during training, coupled with knowledge transfer perception for missing modalities during inference.

This paper presents an integrated framework, which is sequentially unfolded in Section 3, as illustrated in Figure 1.

The core workflow comprises the following steps: First, utilizing the pre-trained image and point cloud networks described in Section 3.2 to freeze and extract high-level RGB and 3D features, thereby providing consistent input for subsequent distillation and memory construction. Subsequently, in Section 3.3, a teacher model generates bimodal “hallucinated” soft labels. Under a training scheme comprising 40% bimodal data, 30% RGB-deficient data, and 30% point cloud-deficient data, the student model learns robust joint representations guided by a cross-entropy plus KL divergence distillation loss. Anomalous data consist of samples containing real industrial defects such as surface scratches, dents, cracks, contaminations, or structural misalignments. Subsequently, in Section 3.4.1, based on the normal sample features extracted by the student model, orthogonal random projection is employed for dimensionality reduction. K-means clustering selects representative prototypes to construct a compact and efficient normal feature memory bank. Finally, in Section 3.4.2, anomaly scores are computed via pixel-level and image-level nearest neighbour distance matching against the memory bank. A single-class SVM enables two-tier decision fusion, achieving high-precision detection and localisation of industrial defects. Through this interlinked modular design, our method strikes a balance between detection accuracy and operational efficiency, while significantly enhancing model robustness against scenarios that are modality-deficient.

### 3.2. Integrated Framework for Cross-Modal Pre-Training and Elastic Inference Under Modal Deficiency

Building upon recent advancements such as the multimodal 3D detection model M3DM [30], the supervised Transformer framework DINO [32], and the point cloud masked autoencoder Point-MAE, pre-trained Transformers demonstrate exceptional performance in distinguishing differential features between normal and anomalous data. Given their proficiency in capturing data transformation relationships, this paper first employs them as deep feature extractors ($\left(\Phi^{2D},{\;\Phi }^{3D}\right)$) to obtain high-level representations suitable for data compression and storage. Specifically, multimodal data input to the feature extractor is denoted as ${\;X}^{2D}$ and ${\;X}^{3D}$, while the extracted output features are denoted as ${\;Z}^{2D}$ and ${\;Z}^{3D}$.

#### 3.2.1. RGB Feature Extraction from

To ensure stable feature extraction during pre-training, basic requirements were defined for the input RGB images. A minimum resolution of 224 × 224 pixels was adopted, consistent with the configuration of vision transformers such as DINO, which balances computational efficiency with the retention of fine-grained texture and defect details. Therefore, in industrial settings, we recommend imaging sensors with at least HD resolution (≥1280 × 720) and adequate illumination to minimise overexposure and specular reflections. These conditions set the lower bound for acceptable image quality and support reproducibility across diverse sensor sources.

For a given RGB image $\;X^{2D}\;\in \;R^{H\;\times \;W\;\times \;3}$, we employ the pre-trained image feature extractor $\;F_{C}$ to map it into a low-dimensional semantic space, generating feature maps.

(1) $$\Phi^{2D}\left(X^{2D}\;\right)\in R^{h\times w\times D_{1}},$$

where  h and  w denote the spatial dimensions after downsampling, and $\;D_{1}$ represents the feature dimension. To achieve cross-modal alignment with point cloud features, bilinear upsampling is applied to this feature map, yielding the aligned feature representation

(2) $$Z^{2D}=\mathrm{UP}\left(\Phi^{2D}\left(X^{2D}\right)\right)\in R^{2h\times 2w\times D_{1}},$$

#### 3.2.2. Three-Dimensional Point Cloud Feature Extraction

To efficiently process large-scale 3D point cloud data and effectively mitigate feature information loss, this paper proposes an adaptive downsampling algorithm based on a voxel-based dynamic resolution adjustment strategy. This method comprises two core modules: dynamic voxel resolution adjustment and adaptive downsampling. Working synergistically, they adaptively adjust the sampling scale to accommodate variations in local point density while precisely preserving critical geometric edge information within the point cloud. This achieves an optimal balance between data simplification and feature fidelity.

For the input point cloud

(3) $$P={\left\{p_{i}\in R^{3}\right\}}_{i=1}^{N},$$

Given a structured point cloud, its inherent sparsity and non-uniform distribution must be carefully considered. To address this, farthest point sampling (FPS) [33] is applied to choose N well-spaced centroids, which act as reference points for forming local neighborhoods through K-nearest neighbor (KNN) search from the original M points. These N subsets are subsequently forwarded to the feature extractor $\;X^{3D}$, which converts them into latent feature embeddings with dimension $N\times d_{2}.$ In practice, following PointNet++, a common setting is to sample 1024 centroids at the input layer, while larger-scale scene segmentation tasks typically adopt 4096 points to ensure sufficient coverage. However, traditional fixed voxel sizes often fail to balance global consistency with local detail when handling regions exhibiting pronounced local density variations. To overcome this limitation, we propose a dynamic voxel segmentation strategy that adaptively adjusts voxel size based on local point density.

To perform spatial voxelisation of the point cloud, we first compute the maximum and minimum values of the point cloud in the  x, y, and  z directions, denoted respectively as

$$X_{\max},Y_{\max},Z_{\max}\;\mathrm{and}\;X_{\min},Y_{\min},Z_{\min},$$

Let

(4) $$l_{x}=X_{\max}-X_{\min},\;l_{y}=Y_{\max}-Y_{\min},\;l_{z}=Z_{\max}-Z_{\min},$$

The maximum voxel block Ω encompassing the entire point cloud can be constructed, with edge lengths $\left\{l_{x},l_{y},l_{z}\right\}$ in each direction. Within this maximum voxel block, we subdivide the three-dimensional space to generate a series of initial voxel cells $\left\{v\right\}$ for subsequent adaptive segmentation.

For each voxel unit v, denote its internal point set as $\;P_{v}$, the number of points as $\left|v\right|$, and the voxel volume as $\mathrm{vol}\;\left(v\right)$. We define the local point density

(5) $$D\left(v\right)=\frac{\left|v\right|}{\mathrm{vol}\left(v\right)},$$

Based on this, we construct the voxel size function

(6) $$s\left(v\right)=s_{min}+\left(s_{max}-s_{min}\right)\cdot \exp\left(-\alpha D\left(v\right)\right),$$

where $s_{\max}$ and $s_{\min}$ denote the upper and lower bounds of the voxel size, respectively, and α>0 is the adjustment parameter. This strategy ensures smaller voxels are employed in dense regions to capture fine details, whilst larger voxels are used in sparse areas to enhance sampling efficiency.

Following adaptive sampling, for each set of points $P_{v}$, within a voxel, we construct local geometric features reflecting the point cloud’s shape and edge information. First, for each point $p_{i}\in P_{v}$, we compute the covariance matrix using its  K neighbourhood set $N\left(p_{i}\right)$:

(7) $$C\left(p_{i}\right)=\frac{1}{\left|N\left(p_{i}\right)\right|}\sum_{q\in N\left(p_{i}\right)}\;(q-\overset{⃐}{q})(q-\overset{⃐}{q}{)}^{⊤},$$

where

(8) $$\overset{⃐}{q}=\frac{1}{\left|N\left(p_{i}\right)\right|}\sum_{q\in N\left(p_{i}\right)}\;q,$$

By performing an eigenvalue decomposition on $C\left(p_{i}\right)$, the eigenvector corresponding to the smallest eigenvalue is taken as the normal vector $\;n\left(p_{i}\right)$ for point $p_{i}$. Based on this, we define the local edge response metric:

(9) $$E\left(p_{i}\right)=\frac{1}{\left|N\left(p_{i}\right)\right|}\sum_{q\in N\left(p_{i}\right)}\;arccos\left(n{\left(p_{i}\right)}^{⊤}n(q)\right),$$

This metric exhibits lower values in flat regions while significantly increasing at edges or areas of structural discontinuity, thereby providing effective guidance for subsequent edge preservation.

To smoothly map the sparsely extracted local features back onto the entire point cloud, we employ an improved inverse distance weighting (IDW) interpolation method. Let the neighbourhood sampling set of the point to be interpolated be denoted as$\;P_{p}$, and the following equation gives its feature vector $\;f\;\left(p\right)$:

(10) $$f\left(p\right)=\frac{\sum_{q\in P_{p}}\;\;w(p,q)f(q)}{\sum_{q\in P_{p}}\;\;w(p,q)},$$

where the weighting function simultaneously accounts for spatial distance and normal vector similarity, defined as

(11) $$w\left(p,q\right)=\exp\left(-\frac{\|p-q{\|}^{2}}{\sigma_{d}^{2}}-\frac{\|n\left(p\right)-n\left(q\right){\|}^{2}}{\sigma_{n}^{2}}\right),$$

$\sigma_{d}$ and $\sigma_{n}$ where they represent the standard deviation parameters for distance and normal vector similarity, respectively. This refinement not only ensures spatial smoothness but also enhances feature discrimination in edge regions.

Finally, through average pooling, we obtain the downsampled feature map $\;R_{PC}\in R^{2H_{f}\times 2W_{f}\times d_{2}}$. By merging the detected edge point set with the downsampled centroid point set, the final point set is formed, effectively preserving key geometric features of the point cloud while significantly reducing computational overhead.

Through the aforementioned design, this module achieves density-adaptive sampling and structure-aware feature extraction for point cloud data, subsequently restoring global features via geometry-guided interpolation. Experimental validation demonstrates that this method accurately preserves local geometric structures and edge information while ensuring data reduction efficiency, providing substantial support for subsequent feature fusion and anomaly detection.

### 3.3. Cross-Distillation Mode

Industrial data acquisition environments are complex and variable, frequently resulting in missing or damaged modalities. This causes traditional multimodal models to rapidly degrade in performance during inference. Therefore, we propose a cross-modal knowledge distillation method based on a teacher-student framework. This enables the model to fully utilise all available data during training, achieving stable and accurate anomaly detection during inference even when certain modalities are missing.

Having acquired features from each modality, we describe our cross-modal training strategy. We propose a teacher-pupil model capable of fully utilising all training samples (even those with missing modalities), while ensuring stable anomaly detection during testing, even when only partial modalities are provided. The core idea is to train teacher models separately for each modality (using all available data for that modality), then use the knowledge from these teacher models to guide the training of a multimodal student model. This ensures that even if a sample lacks a particular modality, it can still provide valuable information for training the student model.

**Figure 2:**
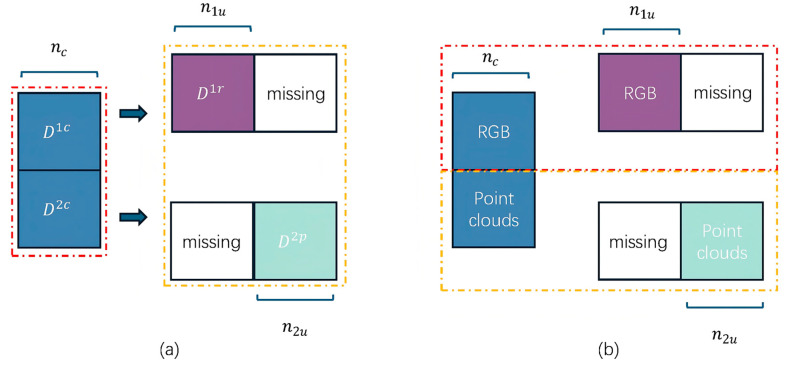
(**a**) The dataset comprises two categories: complete bimodal samples and single-modal samples only. (**b**) Sample selection strategy for constructing the dual-teacher model: samples within the red dashed box are used to train the first single-modal teacher model, while those within the yellow dashed box are used to train the second single-modal teacher model. This ensures that each teacher maximizes the utilization of information specific to their dedicated modality.

First, train two monomodal teacher models using all available data. Represent multimodal samples as $\left\{D^{1c}\in \right.\left.R^{2H_{f}\times 2W_{f}\times d_{1}},\;D^{2c}\in R^{2H_{f}\times 2W_{f}\times d_{2}},d^{c}\in R^{n_{c}}\right\}$. RGB samples are denoted as $\left\{D^{1r}\in R^{2H_{f}\times 2W_{f}\times d_{1}},d^{1u}\in R^{n_{1r}}\right\}$, while 3D point cloud samples are represented as $\left\{D^{2p}\in R^{2H_{f}\times 2W_{f}\times d_{2}},d^{2p}\in \right.\left.R^{n_{2p}}\right\}$. Train the two monomodal teacher models, respectively:

Figure 2a illustrates the data structure. Samples within the blue dashed box possess complete modalities, while those within the yellow dashed box contain only one modality.

(12) $$L_{{Te}_{1}}\left(\phi_{1}\right)=\underset{\phi_{1}}{min}\;\sum_{i=1}^{n_{1}}\;\;H\left(\sigma \left(g_{1}\left(D_{i}^{1};\phi_{1}\right)\right),y_{i}\right),$$

(13) $$L_{{Te}_{2}}\left(\phi_{2}\right)=\underset{\phi_{2}}{min}\;\sum_{i=1}^{n_{1}}\;\;H\left(\sigma \left(g_{2}\left(D_{i}^{2};\phi_{1}\right)\right),y_{i}\right),$$

where  H denotes the cross-entropy loss, $\sigma \left(\cdot \right)$ represents the Softmax function, and $\;n_{1}=n_{c}+n_{1u},\;n_{2}=n_{c}+n_{2u}$.

We then use these two teachers to annotate samples in $\left\{D^{1c},D^{2c}\right\}$. The log for the i-th sample is

(14) $$z_{i}^{1}=Te_{1}\left(D_{i}^{1c};\phi_{1}\right),\;z_{i}^{2}=Te_{2}\left(D_{i}^{2c};\phi_{2}\right),$$

where $z_{i}^{j}$ denotes the log score assigned by teacher j to the i-th sample.

Employing cross-modal knowledge distillation, we train a multimodal student model S that takes images and point clouds (where available) as input and outputs anomaly predictions. The student model is designed to fuse information from both modalities, enabling it to outperform single-modal teachers while adapting to diverse input scenarios. We construct S using a multimodal deep neural network (M-DNN) [34] with a dual-branch architecture, where one branch processes image features and the other handles point cloud features. If the M-DNN is trained following the approach in [35], that is, utilizing only samples with complete modalities {$D^{1c},\;D^{2c}$, $d^{c}$}, the effective training set is restricted to $n_{c}$. When $n_{c}$ is substantially smaller than $\;n_{1}$ and $n_{2}$, a considerable portion of potentially valuable information is discarded. As a result, the number of available training samples becomes insufficient, which may hinder the learning capacity and generalization ability of the model. Therefore, we propose to train the M-DNN by distilling knowledge from two teacher models, $L_{{\mathrm{Te}}_{1}}\left(\phi_{1}\right)$ and $L_{{\mathrm{Te}}_{2}}\left(\phi_{2}\right)$, which are pretrained on substantially larger datasets. Although the classification performance of each teacher may be limited due to their reliance on a single modality, they nevertheless capture modality-specific discriminative patterns. By providing this specialized expertise to the student, the teachers guide it toward learning a more comprehensive multimodal representation. Through this process, the student can effectively integrate complementary knowledge from both modalities, thereby achieving improved performance beyond what unimodal supervision alone can offer, see Algorithm 1.
**Algorithm 1:** Algorithm of the proposed method for two modalities.$\mathrm{Inputs}:\;D^{1c},\;D^{2c}$, $d^{c}$, $D^{1r}$, $d^{1u}$, $D^{2p},\;d^{2p}$, α, β for each training iteration do    Train teacher model $L_{{\mathrm{Te}}_{1}}$ with {[$D^{1c}$, $D^{1r}$], [$d^{c}$, $d^{1u}$]} end for for each training iteration do    Train teacher model $L_{{\mathrm{Te}}_{2}}$ with {[X2c, X2u], [$d^{c}$,$d^{2p}$]} end for Label the samples to train student model with the teachers with Equation (14). for each training iteration do   Train student model with Equation (15) end for

We denote the student network as $S_{\theta }\left(\cdot \right)$, where  θ represents its learnable parameters. The proposed method is trained by minimising the following global objective function:

(15) $$\underset{\theta }{min}\;L(\theta )=\sum_{i=1}^{n_{c}}\;\left[L_{cls}\left(D_{i}^{1},D_{i}^{2},y_{i};\theta \right)+\alpha L_{d_{1}}\left(D_{i}^{1},D_{i}^{2},y_{i};\theta ,T_{e_{1}}\left(\phi_{1}\right)\right)+\beta L_{d_{2}}\left(D_{i}^{1},D_{i}^{2},y_{i};\theta ,T_{e_{2}}\left(\phi_{2}\right)\right)\right],$$

where α and β are hyperparameters regulating the extent to which the student model acquires knowledge from the two teacher models. Larger values indicate the student network requires more information from the corresponding teacher model.

The classification loss $\;L_{\mathrm{cls}}$ is defined in cross-entropy form to measure the discrepancy between the student network’s predictions and the true labels:

(16) $$L_{\mathrm{cls}}\left(D_{i}^{1},D_{i}^{2},y_{i};\theta \right)=H\left(\sigma \left(S_{\theta }\left(D_{i}^{1},D_{i}^{2}\right)\right),y_{i}\right),$$

where $H\left(\cdot \right)$ denotes cross-entropy and $\sigma \left(\cdot \right)$ represents the softmax function.

To further enhance the student network’s performance, we conduct knowledge distillation from two teacher models: $\;T_{e_{1}}\left(\phi_{1}\right)$ and $T_{e_{2}}\left(\phi_{2}\right)$. Specifically, the distillation loss measures the distributional difference between the student network’s output and the teacher models’ outputs using Kullback–Leibler divergence (KL divergence). Let the output vectors of the teacher models on sample $\left(D_{i}^{1},D_{i}^{2}\right)$ be denoted as $z_{i}^{1}$ and $z_{i}^{2}$, respectively. The distillation loss is then defined as follows:

(17) $$L_{d_{1}}\left(D_{i}^{1},D_{i}^{2},y_{i};\theta ,T_{e_{1}}\left(\phi_{1}\right)\right)=F_{\mathrm{KL}}\left(\sigma_{T}\left(S_{\theta }\left(D_{i}^{1},D_{i}^{2}\right)\right),\sigma_{T}\left(z_{i}^{1}\right)\right),$$

(18) $$L_{d_{2}}\left(D_{i}^{1},D_{i}^{2},y_{i};\theta ,T_{e_{2}}\left(\phi_{2}\right)\right)=F_{\mathrm{KL}}\left(\sigma_{T}\left(S_{\theta }\left(D_{i}^{1},D_{i}^{2}\right)\right),\sigma_{T}\left(z_{i}^{1}\right)\right),$$

where $\sigma_{T}\left(\cdot \right)$ denotes the softmax function with temperature coefficient T, employed to smooth the output distribution while preserving greater dark knowledge.

**Figure 3:**
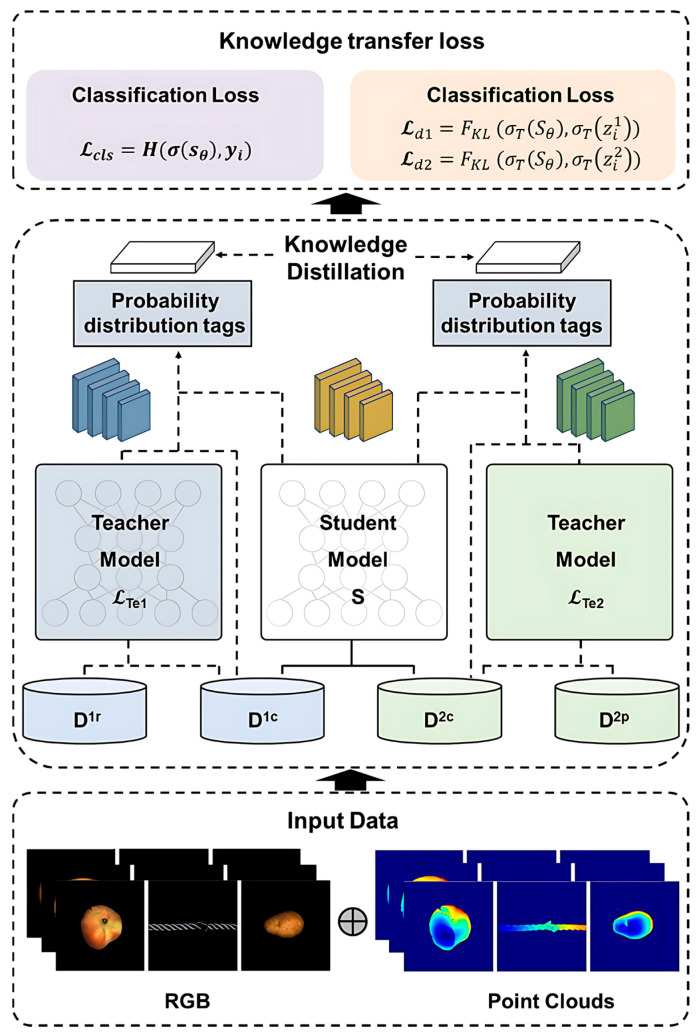
We first pre-train the teacher network on a full dataset containing both complete and missing modality samples, enabling it to learn robust feature representations across both multi-source information and incomplete input scenarios. Consequently, the student network effectively inherits the teacher network’s cross-modal representation capabilities even when receiving only partial modality inputs, achieving high-precision anomaly detection.

Through this training process, the student model S learns a fused representation that fully utilises information from both modalities and has encountered scenarios where one modality is missing during training (via corresponding teacher guidance). Consequently, during inference, the student model can reliably predict anomalies regardless of whether the input is bimodal or unimodal. Figure 2 illustrates the overall structure of the teacher-student distillation framework, see Figure 3.

### 3.4. Unsupervised Feature Fusion

Inference Phase: For any input sample (potentially containing only RGB or point cloud data), the trained student model first extracts its multimodal feature representation. The extracted features are then matched against a pre-constructed normal feature memory bank using nearest neighbour matching, calculating anomaly scores at both image and pixel levels. Finally, a one-class SVM discriminator outputs anomaly detection results (including whether the entire image is anomalous and the pixel-level anomalous regions).

#### 3.4.1. Kernel Selection for Memory Repository Construction

Reference feature library methods quantify abnormality by comparing test sample features with those in a normal sample feature library. Whilst directly storing all normal sample features is feasible, it proves prohibitively costly (requiring substantial storage space and time-consuming matching). Consequently, we require constructing a feature memory bank that is both comprehensive and efficient: on one hand, covering the diversity of normal data as fully as possible to maintain accuracy; on the other, eliminating redundant features to reduce storage and computational overhead. Thus, we aim to build a compact memory bank that encompasses normal data diversity whilst discarding redundant information.

To effectively address this challenge, we employ a clustering algorithm.

The fundamental approach involves partitioning the original feature space into k clusters (where k represents the desired core set size), then selecting one representative point from each cluster as an element within the memory repository. Clustering methods naturally group similar features and extract representative samples from each cluster.

Compared to directly storing all training features, our memory repository requires retaining only k core features, substantially reducing storage and comparison costs (complexity decreases from O(N) to O(k)). Simultaneously, clustering ensures that these core features cover the feature space of normal data as maximally as possible.

Specifically, given a feature set $S\in R^{P\times d}$ (containing  P feature vectors of  d dimensions), we employ the following steps:

1. Apply the k-means clustering algorithm to partition S into k clusters $\left\{C_{1},C_{2},\ldots ,C_{k}\right\}$
2. For each cluster $C_{i}$, select the sample closest to the cluster centre $\mu_{i}$ as its representative:
  (19) $$p_{i}=arg\;\underset{p_{j}\in C_{i}}{min}\;D_{c}\left(p_{j},\mu_{i}\right),$$
  where $\;D_{c}$ is the distance metric function. This metric should align with that used for calculating anomaly scores to ensure consistency between feature selection and anomaly detection processes.
3. All selected representative points $\left\{p_{1},p_{2},\ldots ,p_{k}\right\}$ form the final core set $M_{\mathrm{coreset}}$

In determining the number of clusters k, we adopted the Elbow Method, which calculates the Within-Cluster Sum of Squares (WCSS) for different values of k and plots the curve. As k increases, the WCSS decreases progressively, but after a certain point, the rate of decrease slows down markedly, resulting in an “elbow” shape in the curve. The corresponding value of k at this elbow is regarded as the optimal choice. At this point, adding more clusters yields only marginal improvements. By applying this criterion, we ensure sufficient coverage of the diversity of normal data features while avoiding unnecessary redundancy in storage and computation, thereby achieving a balance between accuracy and efficiency. Consequently, the value of k used in our experiments was determined based on this principle.

However, as the sample size increases, similarity calculations among high-dimensional features remain highly computationally intensive. To address this, we introduce Orthogonal Random Projection (Li et al., 2022) [36] to reduce feature dimensions. This method preserves relative distance relationships between features, significantly accelerating computation while maintaining acceptable representation quality. Experiments demonstrate that applying this technique increases feature extraction speed by approximately 3.5 times, while incurring a loss of less than 2% in detection accuracy.

Following the aforementioned teacher-student training, we obtained a student model capable of extracting fused features. Subsequently, we utilise this model to extract features from normal samples and construct a reference memory bank for anomaly detection.

#### 3.4.2. Decision Layer Fusion

Previous anomaly detection approaches predominantly calculated singular anomaly scores at the image level or focused solely on pixel-level heatmaps. Our method, however, integrates both image-level and pixel-level assessments, employing a class-based Support Vector Machine (SVM) to fuse these two information streams. This dual-level decision fusion is anticipated to enhance detection reliability—ensuring overall accuracy at the image level while providing precise localisation at the pixel level.

During inference, the model calculates anomaly scores by comparing each real or generated feature map ($F_{PC}$ and $\;F_{RGB}$) against the corresponding memory repository. Two single-class top-port vector machines employing stochastic gradient descent techniques $C_{C}$ and $C_{s}$ serve as the decision layer fusion for image classification and segmentation:

(20) $$c=C_{c}\left(\alpha \psi \left(F_{PC},M_{PC}\right),\beta \psi \left(F_{RGB},M_{RGB}\right)\right),$$

(21) $$s=C_{s}\left(\alpha \phi \left(F_{PC},M_{PC}\right),\beta \phi \left(F_{RGB},M_{RGB}\right)\right),$$

C and S denote the final prediction scores for image classification and pixel segmentation, respectively. To balance the average anomaly scores across modalities, correction factors  α and  β are introduced ψ and ϕ represent the anomaly scores for classification and segmentation, formulated as (Roth et al., 2022) [37]:

(22) $$\begin{array}{c} \psi \left(F,M\right)=D_{d}\left(F^{\left(i,j\right),*},m^{*}\right) \end{array},$$

(23) $$\phi (F,M)=\left\{\underset{m\in M}{min}\;D_{d}\left(F^{i,j},m\right),\;\mathrm{for}\;F^{i,j}\in F\right\},$$

$D_{d}$ is the distance metric. The anomaly score for an image is determined by the maximum distance between its features and the most similar features in its memory bank:

(24) $$F^{(i,j),*},m^{*}=arg\;\underset{F^{\left(i,j\right)}\in F}{max}\;arg\;\underset{m\in M}{min}\;D_{d}\left(F^{i,j},m\right),$$

During inference, to fully leverage knowledge acquired through multimodal training, we employ a memory bank matching strategy to assign anomaly scores to input feature maps. Specifically, let feature maps extracted from point clouds and RGB images be denoted as $\;X_{P}$ and $X_{R}$, respectively, with corresponding memory banks $\;M_{P}$ and $\;M_{R}$. To balance scoring scale differences across modalities, we introduce correction factors  γ and δ. Ultimately, two One-Class Support Vector Machine (One-Class SVM) decision trees are employed to determine image classification and pixel-level segmentation, respectively. Their fusion formula is defined as:

(25) $$c=f_{c}\left(\gamma \cdot S\left(X_{P},\;M_{P}\right),\delta \cdot S\left(X_{R},\;M_{R}\right)\right),$$

(26) $$s=f_{c}\left(\gamma \cdot S\left(X_{P},\;M_{P}\right),\delta \cdot S\left(X_{R},\;M_{R}\right)\right),$$

where $f_{c}\left(\cdot \right)$ and $f_{s}\left(\cdot \right)$ represent the classification and segmentation decision functions, respectively (both implemented using One-Class SVM optimised by stochastic gradient descent), while c and s denote the overall image anomaly score and the anomaly response for each pixel, respectively.

To construct the scoring function, we derive two anomaly scoring metrics based on the distance between features and the memory repository:

1. Classification Anomaly Score

Define the function $S\left(X,M\right)$ to quantify the most “anomalous” local features within the feature map X. Its construction proceeds as follows: First, identify the most difficult-to-match feature within X that satisfies the following condition:

(27) $$x^{*}=arg\;\underset{x\in X}{max}\;\underset{m\in M}{min}\;d(x,m),$$

Then, let the most similar memory entry corresponding to$\;x^{*}$ be

(28) $$m^{*}=arg\;\underset{m\in M}{min}\;d\left(x^{*},m\right),$$

Finally, the classification anomaly score is defined as

(29) $$S\left(X,M\right)=d\left(x^{*},m^{*}\right),$$

This score reflects the maximum degree of mismatch between the input sample’s overall feature distribution and that of the normal sample database.

2. Segmentation Anomaly Score

To characterise pixel-level anomalies, we define the function $\;A\;\left(X,\;M\right)$ to compute the anomaly response at each position $\left(i,j\right)$ in the feature map, i.e.,

(30) $$A(X,M)(i,j)=\underset{m\in M}{min}\;d(X(i,j),m),$$

This definition represents the distance between the input feature at position $\;\left(i,\;j\right)$ and the most similar feature in the memory database. A larger distance indicates a higher likelihood of an anomaly at that position.

In summary, by computing anomaly scores for both modalities and applying scale normalisation via correction factors, the decision-layer fusion module comprehensively utilises multimodal information across classification and segmentation tasks. This enhances the overall accuracy and robustness of anomaly detection.

## 4. Experiments

### 4.1. Experimental Details

#### 4.1.1. Dataset and Evaluation Metrics

Three-dimensional industrial anomaly detection remains in its infancy, with the MVTec-3D AD dataset [3] serving as the standard dataset for this task. We conducted experiments on this dataset. The MVTec-3D AD dataset comprises 10 categories, with 2656 training samples and 1137 test samples. All samples were acquired using industrial 3D sensors employing structured light technology for 3D scanning, with positional information stored as a 3-channel tensor representing x, y, and z coordinates. Additionally, each point in the dataset records RGB information aligned with its corresponding three-dimensional point cloud (PC). As all samples originate from the same viewpoint, the RGB information for each sample can be stored as a single image, thereby providing multimodal data encompassing both RGB and three-dimensional positional information for each sample. Each sample in this dataset comprises a colour point cloud image, providing point cloud coordinates in 3D space alongside its corresponding RGB image.

Recent multimodal industrial anomaly detection research (e.g., Bergmann et al., 2022) [4] predominantly utilises the MVTec-3D AD dataset, which records aligned point clouds and RGB images of real objects. Within each category, objects may exhibit 3 to 5 defects with pixel-level accuracy. The 3D sensor scans objects using structured light technology to acquire point cloud data, which is combined with RGB images to provide multimodal input for model training. Additionally, we evaluated our method on the purely synthetic RGB + 3D IAD dataset Eyecandies [38]. During evaluation, the performance of image-level and pixel-level anomaly detection was assessed using the area under the receiver operating characteristic curve (I-AUROC and P-AUROC) and the area under the probability overlap curve (AUPRO), respectively. Compared to P-AUROC, AUPRO places greater emphasis on the overlap between predicted results and each connected segment in the ground truth, effectively mitigating the impact of anomaly size on detection performance.

#### 4.1.2. Data Preprocessing

This study conducted experiments in the Ubuntu 22.04 operating system environment using an NVIDIA RTX 3090 graphics card (manufactured by NVIDIA Corporation, Santa Clara, CA, USA). The environment dependencies and their corresponding versions are shown in Table 1.

To mitigate the detrimental impact of background artefacts on classifier performance, the 3D point cloud preprocessing stage first employs the RANSAC algorithm for robust fitting of background planes within the point cloud. All points within 0.005 meters of this plane are subsequently removed, effectively eliminating irrelevant planar noise. This operation not only significantly reduces computational overhead during 3D feature extraction in both training and inference phases but also minimizes background interference during anomaly detection, thereby enhancing detection accuracy. Finally, to maintain consistent input dimensions with the subsequent multimodal feature extraction network, which employs adaptive downsampling via DINO and a voxel resolution dynamic adjustment strategy, the coordinates of the background-removed point cloud and the corresponding RGB images are uniformly resampled to 224 × 224 pixels. This ensures seamless integration between the preprocessing workflow and model input.

#### 4.1.3. Experimental Implementation Details

For feature extraction, we employed models pre-trained on large-scale datasets: DINO for RGB images, pre-trained on the ImageNet dataset [39]; and an algorithm utilizing a dynamic voxel resolution adjustment strategy for adaptive down-sampling of point cloud data. This combined feature extraction approach references the design outlined in [40]. Specifically, the input RGB images are resized to 224 × 224 × 3, which is the standard input resolution required by the pre-trained DINO vision transformer. Following feature extraction by the pre-trained models, the feature representations are unsampled to a spatial dimension of 56 × 56, yielding a feature map with 768 channels. For point cloud data, we further introduced a dynamic voxel resolution adjustment mechanism based on local density to adaptively down-sample different regions: finer voxels are allocated in high-density areas, while larger voxels are employed in sparse regions. This approach minimises redundant points while maximising the preservation of geometric detail. Subsequently, structure-aware feature extraction is performed on the point set within each voxel unit: a neighbourhood point cloud is constructed via k-nearest neighbour search, its covariance matrix is computed, and principal normal vectors are obtained through feature decomposition. Edge response metrics are defined based on normal vector differences to identify and preserve geometric edges within the point cloud. Subsequently, utilising an enhanced inverse distance weighting [41] method, the sparsely extracted structure-aware features are smoothly interpolated onto a 56 × 56 spatial grid identical to the RGB feature map. The channel count is expanded to 768 dimensions to achieve cross-modal alignment. Finally, at the decision layer, we feed the aligned RGB and point cloud features in parallel to two One-Class SVMs. Optimization is performed using stochastic gradient descent [42] with a learning rate of 1.3 × 10^−4^ over 900 iterations, constructing a multimodal discriminative model that supports both image-level classification and pixel-level segmentation.

For multimodal cross-modal distillation, to simulate incomplete modalities in real-world scenarios, 40% of samples were randomly selected according to a discrete uniform distribution, ensuring that each sample had an equal probability of being chosen to retain both modalities. For the remaining samples, one modality was independently removed with a 30% dropout rate. All teacher networks employed a three-layer fully connected architecture, with hidden node counts optimised via validation sets within the range {16, 32, 64, 128, 256}. The student network within the TS (Teacher–Student) framework and the multimodal DNN (M-DNN) baseline retained identical network topologies to those used in the data generation phase, with weights initialized from scratch and trained anew. To prevent bias, the two sets of weight coefficients α and β in the distillation loss are consistently enforced to be equal, with their optimal values sought within {0.1, 0.2, …, 0.9}; the temperature parameter T is selected from five levels: {1, 5, 10, 15, 20}.

### 4.2. Comparative Experiments

Table 2, Table 3, Table 4 and Table 5 present our method’s category-wise and average performance comparisons for I-AUROC and AUPRO metrics across 10 classes on the MVTec 3D-AD dataset. To comprehensively evaluate our approach, we contrast it with existing methods encompassing single-RGB, single-3D point cloud, depth image, and multimodal approaches combining RGB with either point cloud or depth image. Experimental results demonstrate that, compared to methods utilising solely RGB data, our approach achieves a 2.1% improvement in I-AUROC and a 0.7% improvement in AUPRO relative to the best single-modality methods. When compared to single 3D point cloud approaches, our method achieves a 0.3% improvement in I-AUROC and a 0.4% improvement in AUPRO. Compared to state-of-the-art methods based on RGB plus point cloud or depth images, our approach achieves optimal performance with I-AUROC and AUPRO scores of 0.938 and 0.947, respectively. This represents a 2.2 percentage point improvement in I-AUROC and a 0.5 percentage point gain in AUPRO over the current best methods. These results fully validate the effectiveness and superiority of cross-modal knowledge distillation in industrial anomaly detection tasks.

The point cloud simplification ratio (PCR) is generally considered an important quantitative indicator for measuring the down-sampling effect of point cloud maps. Its calculation method is shown in the following formula [50]. This indicator can intuitively reflect the change ratio of the number of point clouds before and after down-sampling, thus providing a quantitative basis for the trade-off analysis between point cloud compression efficiency and geometric fidelity. In existing research on point cloud processing and map construction, the simplification ratio has been widely used to evaluate the effectiveness of different down-sampling strategies.

(31) $$R=\frac{\mathrm{NUM}-{\mathrm{num}}_{d}}{\mathrm{NUM}},$$

where NUM is the total number of points in the original point cloud, ${\mathrm{num}}_{d}$ is the total number of points in the down-sampled point cloud, and R is the point cloud simplification ratio.

To evaluate the effectiveness of our proposed dynamic down-sampling algorithm, we conducted comparative experiments against several conventional down-sampling strategies, including voxel-based, uniform, random and farthest point sampling (FPS). The experiments were performed on raw point clouds containing 216,942 points. Table 6 summarizes the comparative analysis, demonstrating that our method achieves an excellent balance between simplification ratio (91.89%) and processing speed (0.0139 s), thereby highlighting its efficiency and suitability for real-time applications. Specifically, the algorithm reduces the number of points from 216,942 to 17,537 while preserving essential structural details, without requiring the extensive manual parameter tuning demanded by many traditional approaches. This adaptability not only simplifies the down-sampling process but also enhances its practicality and applicability in diverse industrial scenarios.

To further validate that the observed performance gains are not attributable to random fluctuations, we conducted paired-sample *t*-tests comparing our method with representative baselines across the ten categories of the MVTec 3D-AD dataset. The results in Table 7, Table 8 and Table 9 demonstrate that our method achieves significantly higher I-AUROC scores than several representative approaches, including FPFH (t = 3.969, *p* < 0.05), AST (t = 2.191, *p* < 0.05), BTF (t = 2.251, *p* < 0.05), Shape-Guided (t = 0.694, *p* < 0.05), and M3DM (t = 2.045, *p* < 0.05). For AUPRO, our model also significantly surpasses the single point cloud baseline (t = 4.586, *p* < 0.05), whereas the difference with the single-RGB model is not statistically significant (t = 2.357, *p* > 0.05). Importantly, the *t*-test results obtained from Table 3, Table 4 and Table 5 reveal patterns consistent with those observed in Table 2: our method exhibits statistically significant improvements over the majority of baselines, with only a few comparisons against unimodal RGB models showing non-significant differences. Moreover, the ablation study confirms that dual-modality RGB + point cloud variants substantially outperform unimodal baselines (e.g., Dual-RGB vs. Single-RGB: t = 12.366, *p* < 0.05 for I-AUROC; t = 3.306, *p* < 0.05 for AUPRO), underscoring the effectiveness of multimodal knowledge transfer. Collectively, these findings provide compelling statistical evidence that the improvements of our approach are not only numerically superior but also robust, reproducible, and generalizable across datasets, modalities, and evaluation metrics. This reinforces the practical significance of cross-modal knowledge distillation for advancing industrial anomaly detection.

### 4.3. Melting Experiments

We conducted ablation experiments to validate the efficacy of the multi-modal training, few-modal inference (MTFI) workflow, and assessed its generalisation capability for asymmetric performance by swapping feature extractors and datasets. In our experiments, we replaced the original RGB feature extractor, DINO ViT-B/8 [51], with Swin Transformer and DINO ViT-S/8. We substituted the 3D point cloud feature extractor, Point-MAE, with PointNet and PointCNN, and switched the dataset from MVTec 3D-AD to Eyecandies. Experimental results, as summarized in Table 7, demonstrate that the MTFI-based approach significantly outperforms unimodal methods and, in certain scenarios, even surpasses dual-modal RGB-3D point cloud memory-based methods. However, when performing inference using RGB data, the MTFI process does not yield a substantial performance improvement over a single RGB memory. Collectively, these findings further indicate that the MTFI process effectively enhances the utilisation of multimodal training data when performing inference using the principal component (3D point cloud), see Table 10.

### 4.4. Visualisation Results

**Figure 4:**
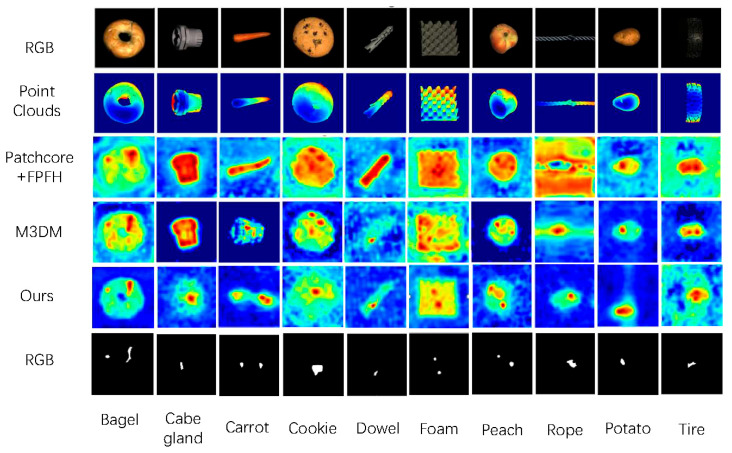
In comparison with existing approaches, the proposed method demonstrates robustness against noise and achieves a more precise delineation of the segmentation region.

In this section, we present a more intuitive visualisation of the segmentation results for all classes in the MVTec-3D AD dataset. As shown in Figure 4, we display the heatmap results for our method and PatchCore + FPFH. Both FPFH and PatchCore utilise multimodal inputs. Compared to the results from PatchCore + FPFH, our method achieves superior segmentation maps.

## 5. Conclusions

This study presents an innovative framework for industrial anomaly detection that integrates cross-modal pre-training, missing-modality-resilient inference, and dynamic voxel down-sampling. The framework explicitly addresses two major types of data incompleteness encountered in industrial inspection: (i) incomplete sensor data within a modality, such as partial or noisy measurements, and (ii) incomplete modalities, where an entire sensing channel becomes unavailable. By jointly solving these challenges and mitigating the computational cost of 3D point-cloud processing, the proposed method achieves state-of-the-art performance on the MVTec 3D-AD dataset. It delivers stable results under single-modal or degraded-data conditions while maintaining high efficiency, providing a practical solution for real-world industrial deployment. These results confirm the framework’s capability to preserve both accuracy and efficiency under varying levels of information loss.

Nevertheless, the robustness of anomaly detection has an intrinsic upper bound: when data corruption or modality loss exceeds a critical threshold, the remaining signals may no longer contain sufficient discriminative information for reliable detection. From an information-theoretic perspective, once the mutual information between the observable input and the underlying anomaly distribution approaches zero, detection becomes theoretically infeasible. Although our cross-modal distillation pushes this boundary further than previous approaches, it cannot compensate for a complete absence of informative content. Future work should empirically and analytically quantify this limit through controlled ablation experiments and mutual-information analysis. This theoretical insight provides valuable guidance for enhancing network robustness and developing adaptive strategies under uncertain sensor availability.

When only intra-modal degradation occurs, the edge-preserving voxelization module can operate independently to improve geometric fidelity. In contrast, under total modality loss, the distilled student network performs inference by leveraging the cross-modal priors learned during training. Beyond these scenarios, future research may extend the framework to additional sensing modalities such as thermal imaging and X-ray, and explore automated selection of optimal modality combinations to further improve precision, efficiency, and robustness in industrial inspection. Overall, this work advances the development of modality-resilient multimodal learning for intelligent manufacturing and related industrial applications.

## Figures and Tables

**Table 1:** List of environmental dependencies.

System Environment	Version
ubuntu	22.04
cuda	12.1
python	3.11
pytorch	2.2.0

**Table 2:** I-AUROC results for MVTec 3D-AD using RGB-based methods.

Method	Bagel	Cable Gland	Carrot	Cookie	Dowel	Foam	Peach	Potato	Rope	Tyre	Mean
FPFH [43]	0.825	0.551	0.952	0.797	0.883	0.582	0.758	0.889	0.929	0.653	0.782
AST [30]	0.881	0.576	0.965	0.957	0.679	0.797	0.990	0.915	0.956	0.611	0.833
BTF [44]	0.938	0.765	0.972	0.888	0.960	0.664	0.904	0.929	0.982	0.726	0.837
Shape-guided [45]	0.983	0.682	0.978	0.998	0.960	0.737	0.993	0.979	0.966	0.871	0.916
M3DM [46]	0.941	0.651	0.965	0.969	0.905	0.760	0.880	0.974	0.926	0.765	0.874
Ours_single	0.834	0.884	0.954	0.929	0.942	0.789	0.869	0.983	0.882	0.872	0.895
Ours_F2F	0.992	0.930	0.971	0.893	0.953	0.893	0.933	0.957	0.951	0.902	0.938

**Table 3:** AUPRO results for MVTec 3D-AD based on RGB method.

Method	Bagel	Cable Gland	Carrot	Cookie	Dowel	Foam	Peach	Potato	Rope	Tyre	Mean
FPFH	0.973	0.879	0.982	0.906	0.892	0.735	0.977	0.982	0.956	0.961	0.924
AST	0.943	0.818	0.977	0.882	0.881	0.743	0.958	0.974	0.950	0.929	0.906
BTF	0.976	0.927	0.979	0.974	0.971	0.884	0.776	0.881	0.959	0.911	0.914
Shape-guided	0.947	0.826	0.977	0.882	0.881	0.767	0.967	0.978	0.947	0.940	0.911
M3DM	0.974	0.871	0.981	0.924	0.898	0.773	0.978	0.983	0.955	0.969	0.931
Ours_single	0.974	0.847	0.973	0.873	0.947	0.826	0.956	0.962	0.942	0.943	0.924
Ours_F2F	0.981	0.889	0.989	0.913	0.934	0.888	0.972	0.972	0.959	0.977	0.947

**Table 4:** I-AUROC results for MVTec 3D-AD based on the 3D method.

Method	Bagel	Cable Gland	Carrot	Cookie	Dowel	Foam	Peach	Potato	Rope	Tyre	Mean
Voxel VM	0.553	0.772	0.484	0.701	0.751	0.578	0.480	0.466	0.689	0.611	0.609
PADiM [47]	0.975	0.775	0.698	0.582	0.959	0.663	0.858	0.535	0.832	0.760	0.764
PatchCore	0.876	0.880	0.791	0.682	0.912	0.701	0.695	0.618	0.841	0.702	0.770
CS-Flow [48]	0.941	0.930	0.827	0.795	0.990	0.886	0.731	0.471	0.986	0.745	0.830
M3DM	0.944	0.918	0.896	0.749	0.959	0.767	0.919	0.648	0.938	0.767	0.850
AST	0.983	0.873	0.976	0.971	0.932	0.885	0.974	0.091	0.832	0.797	0.825
Ours_single	0.932	0.938	0.888	0.739	0.969	0.797	0.920	0.624	0.949	0.771	0.853
Ours_F2F	0.992	0.930	0.971	0.893	0.953	0.893	0.933	0.957	0.951	0.902	0.938

**Table 5:** AUPRO results for MVTec 3D-AD based on the 3D method.

Method	Bagel	Cable Gland	Carrot	Cookie	Dowel	Foam	Peach	Potato	Rope	Tyre	Mean
Voxel VM [49]	0.510	0.331	0.413	0.715	0.680	0.279	0.300	0.507	0.611	0.366	0.471
3D-ST	0.950	0.483	0.986	0.921	0.905	0.632	0.945	0.988	0.976	0.542	0.833
CS-Flow	0.855	0.919	0.958	0.867	0.969	0.500	0.889	0.935	0.904	0.919	0.871
PatchCore	0.901	0.949	0.928	0.877	0.892	0.563	0.904	0.932	0.908	0.906	0.876
PADiM	0.980	0.944	0.945	0.925	0.961	0.792	0.966	0.940	0.937	0.912	0.930
M3DM	0.952	0.972	0.973	0.891	0.932	0.843	0.970	0.956	0.968	0.966	0.942
Ours_single	0.953	0.982	0.969	0.903	0.947	0.860	0.966	0.959	0.969	0.965	0.946
Ours_F2F	0.981	0.889	0.989	0.913	0.934	0.888	0.972	0.972	0.959	0.977	0.947

**Table 6:** Quantitative comparison of different point cloud down-sampling strategies in the proposed framework.

Methods	Number of Down-Sampled Points	Simplification Rate	Time (s)	Parameters
Voxel down-sampling	104,356	0.5169	0.1025	leafsize = 0.01
	5225	0.9759	0.0125	leafsize = 0.05
	1440	0.9934	0.0085	leafsize = 0.1
Random down-sampling	2170	0.9900	0.0143	leafsize = 0.01
	10,847	0.9500	0.0126	leafsize = 0.05
	21,694	0.9000	0.0135	leafsize = 0.1
Uniform down-sampling	2177	0.9899	0.1129	every_k_points = 100
	8687	0.9599	0.3493	every_k_points = 25
	21,694	0.9000	1.0615	every_k_points = 10
FPS	1000	0.9745	34.8717	arget_number_of _triangles = 5000
	5000	0.9490	65.9702	arget_number_of _triangles = 10,000
	10,000	0.9235	109.9000	arget_number_of _triangles =15,000
Our method	17,557	0.9187	0.0139	

**Table 7:** Experimental comparison table of various down-sampling methods.

Pair	Mean Difference	Std. Deviation	Std. Error Mean	95% CI Lower	95% CI Upper	t	df	Sig. (2-Tailed)
FPFH	0.155600	0.123984	0.039207	0.066907	0.244293	3.969	9	0.003
AST	0.104800	0.151272	0.047836	0.066870	0.213014	2.191	9	0.056
BTF	0.064700	0.090896	0.028744	−0.000323	0.129723	2.251	9	0.051
Shape-guided	0.022800	0.103878	0.032849	−0.051510	0.097110	0.694	9	0.505
M3DM	0.063900	0.098830	0.031253	−0.006799	0.134599	2.045	9	0.071
RGB-single	0.043700	0.058631	0.018541	0.001758	0.085642	2.357	9	0.043

**Table 8:** Paired-sample *t*-test results for I-AUROC scores in the ablation study.

Pair	Mean Difference	Std. Deviation	Std. Error Mean	95% CI Lower	95% CI Upper	t	df	Sig. (2-Tailed)
Ours_PC—Single_PC	0.029500	0.052361	0.026180	−0.053818	0.112818	1.127	3	0.342
Ours_RGB—Single_RGB	0.021250	0.035208	0.017604	−0.034773	0.077273	1.207	3	0.314
Dual_RGB—Single_PC	0.027500	0.039408	0.019704	−0.035207	0.090207	1.396	3	0.257
Dual_RGB—Single_RGB	0.042750	0.006397	0.003198	0.032572	0.052928	13.366	3	0.001

**Table 9:** Paired-sample *t*-test results for AUPRO scores in the ablation study.

Pair	Mean Difference	Std. Deviation	Std. Error Mean	95% CI Lower	95% CI Upper	t	df	Sig. (2-Tailed)
Ours_PC—Single_PC	0.012750	0.005560	0.002780	0.003902	0.021598	4.586	3	0.019
Ours_RGB—Single_RGB	0.004750	0.004031	0.002016	−0.001664	0.011164	2.357	3	0.100
Dual_RGB—Single_PC	0.010750	0.026663	0.013332	−0.031677	0.053177	0.806	3	0.479
Dual_RGB—Single_RGB	0.021250	0.012971	0.006486	0.000610	0.041890	3.277	3	0.047

**Table 10:** Ablation study for different feature extractors. The top and bottom of each row are the average I-AUROC and AUPRO.

Feature Extractors	Single PCs	Ours_PCs	Single RGB	Ours_RGB	Dual RGB + Principal Components
Swin Transformer + PointNet	0.832	0.938	0.864	0.938	0.916
	0.919	0.934	0.941	0.940	0.962
Swin Transformer + PointCNN	0.908	0.898	0.893	0.896	0.932
	0.863	0.874	0.905	0.098	0.911
DINO ViT-S/8 + PointNet	0.863	0.882	0.831	0.833	0.869
	0.893	0.912	0.851	0.860	0.891
DINO ViT-S/8 + PointCNN	0.909	0.912	0.863	0.869	0.905
	0.915	0.921	0.883	0.890	0.920

## Data Availability

The original contributions presented in this study are included in the article. Further inquiries can be directed to the corresponding authors.
